# Erythrocyte and Reticulocyte Indices on the LH 750 as Potential Markers of Functional Iron Deficiency

**DOI:** 10.1155/2010/625919

**Published:** 2010-06-16

**Authors:** Eloísa Urrechaga, Luís Borque, Jesús F. Escanero

**Affiliations:** ^1^Hematology Laboratory, Hospital Galdakao—Usansolo, Galdakao, 48960 Vizcaya, Spain; ^2^Department of Pharmacology and Physiology, Faculty of Medicine, University of Zaragoza, Zaragoza, Spain

## Abstract

Reticulocyte hemoglobin content (CHr) and percentage of hypochromic cells (%Hypo) are restricted to the Siemens analysers. The aims of the study were to investigate the correlation of Red cells size factor (RSf) and low Hemoglobin density (LHD%), reported by Beckman-Coulter analysers, with CHr and %Hypo in the assessment of iron status in the presence of inflammation. 381 samples were run on both LH 750 (Beckman-Coulter) and Advia 2120 (Siemens) analysers. Correlation between parameters were calculated and the diagnostic performance of the new parameters was assessed. *Results*. Correlation between RSf and CHr, *r* = 0.85. ROC curve analysis for RSf in the diagnosis of iron restricted erythropoiesis defined as CHr < 28 pg: AUC 0.983; Cutoff 91.1%; Sensitivity 98.8%; Specificity 89.6% Correlation between LHD% and %Hypo, *r* = 0.869. ROC curve analysis for LHD% in the diagnosis of iron deficiency defined by %Hypo >5%: AUC 0.954; Cut off 6.0%; Sensitivity 96.6%; Specificity 83.2% *Conclusions*. RSf and LHD% could be reliable parameters for the study of iron metabolism status.

## 1. Introduction

In iron deficiency anemia (IDA) iron supply depends on the quantity of iron storage in the body, while in functional iron deficiency (iron restricted erythropoiesis) supply depends on the rate of mobilization of iron from the stores. Functional iron deficiency is defined as an imbalance between the iron needs for erythropoiesis and the iron supply, with the latter not maintained at sufficient rate for adequate hemoglobinization of reticulocytes and mature erythrocytes [1].

The diagnosis of iron deficiency or functional iron deficiency is particularly challenging in patients with acute or chronic inflammatory conditions because most of the biochemical markers for iron metabolism are affected by acute phase reaction. This is the case of the anemia of chronic disease (ACD) and the anemia associated to chronic renal failure (CKD).

Serum ferritin, an indicator of iron storage but not of iron supply, is a positive acute phase reactant, while transferrin is a negative acute phase reactant, rendering the calculation of transferrin saturation unreliable in this case [2–4].

For these reasons, an iron deficient erythropoiesis may occur despite that normal serum ferritin and transferrin saturation values and interest have been generated in the use of erythrocyte and reticulocyte parameters, available on the modern analysers based on flow cytometry technology.

Direct consequence of an imbalance between the erythroid marrow iron requirements and the actual supply is a reduction of red cell hemoglobin content, which causes hypochromic mature red cells and reticulocytes. 

Reticulocyte hemoglobin content (CHr) and the percentage of hypochromic red blood cells (%Hypo) reflect iron availability and are reliable markers of functional iron deficiency [5, 6].

The measurement of CHr is a direct assessment of the incorporation of iron into erythrocyte hemoglobin (Hb) and thus an estimate of the recent functional availability of iron into the erythron; due to the life span of the reticulocytes, CHr is a sensitive indicator of iron deficient erythropoiesis [7–9].

The measurement of %Hypo (defined as the percentage of red blood cells with Hb concentration less than 280 g/L) is a sensitive method for quantifying the hemoglobinization of mature red cells. Because of the long circulating life span of mature erythrocytes %Hypo values are related to iron status in the last 2-3 months, and have been recognised as an indicator of iron deficiency [10, 11].

CHr and %Hypo have been used as a diagnostic tool, together with biochemical markers, to distinguish IDA from ACD, and are incorporated to National Kidney Foundation (NKF-K/DOQI) guidelines for the monitoring of recombinant human erythropoietin rHuEPO therapy [12–14].

To date, the measurement of CHr and %Hypo has been restricted to the analysers of a single manufacturer, Siemens (Siemens Medical Solutions Diagnostics, Tarrytown N.Y, USA). A second manufacturer has produced a comparable index, the so-called reticulocyte hemoglobin equivalent (Ret He) generated by the Sysmex XE 2100 analyser (Sysmex Corporation, Kobe, Japan).

Measurements of Ret He provides useful information in diagnosing anemia, iron restricted erythropoiesis, and functional iron deficiency and response to iron therapy during r-HuEPO. Twenty nine pg is the cutoff value that defines deficient erythropoiesis [15, 16]. Ret He correlates with CHr with the same clinical meaning [17]. The new Symex XE 5000 analyser reports the percentages of hypochromic red cells, but few data are already published about this parameter [18].

Beckman Coulter (Beckman Coulter Inc., Miami, Fl, USA) has recently introduced on the LH series analysers two new parameters

Red blood cell Size Factor (RSf) joins together the volume of mature red cells (MCV) and the volume of reticulocytes (MRV), both related to erythropoietic activity and hemoglobinization

(1) $$\text{RSf}=\sqrt{{\text{MCV}}^{*}\text{MRV}}.$$

Compared to the mature erythrocyte population, reticulocytes have a mean volume 15–20 fL greater, they stay in the blood stream for 1–1.5 days, so the measurement of reticulocyte number and cellular characteristics, such as volume, provides real-time data regarding certain aspects of erythropoiesis that can influence the dimensions of red cells, such as iron availability. The examination of both precursors and mature cells provides an opportunity to detect and monitor acute and chronic changes in cellular hemoglobin status, related to cell volume [19].

Low hemoglobin density (LHD%) derives from the traditional mean cell hemoglobin concentration (MCHC), using the mathematical sigmoid transformation

(2) $$\text{LHD\%}=100^{*}\sqrt{1-\left(\frac{1}{\left(1+e^{1.8(30-\text{MCHC})}\right)}\right)}.$$

MCHC is an all-inclusive measure of both the availability of iron over the preceding 90–120 days, and of the proper introduction of iron into intracellular hemoglobin. 

In the same way, LHD% is related to iron availability and the hemoglobinization of the mature red cells.

In this equation defining LHD%, in addition to the standard sigmoid function, a square root is applied to further enhance numerical resolution in the region corresponding to the lower end of %Hypo, to improve the differentiation between the normal and the abnormal among the blood samples having relatively low values of LHD% [20].

The aims of this study were

- to establish the reference range of RSf and LHD% and their values in different types of anemia;
- to evaluate these recently introduced reticulocyte and erythrocyte parameters provided by the LH series analysers in terms of correlation with CHr and %Hypo as well as their diagnostic efficiency assuming CHr < 28.0 pg or %Hypo > 5% to detect iron restricted erythropoiesis and iron deficiency.

## 2. Materials and Methods

### 2.1. Criteria for Selecting the Groups of Patients

Samples from 120 healthy individuals, 72 iron deficiency anemia (IDA), 60 IDA with acute phase response (IDA APR), 71 chronic kidney disease (CKD), and 58 anemia of chronic disease (ACD) were randomly extracted from the routine workload and run sequentially on both LH 750 (Beckman Coulter Inc. Miami, Fl, USA) and Advia 2120 (Siemens Medical Solutions Diagnostics, Tarrytown N.Y., USA), analysers within 6 hours of collection.

Healthy group: 54 male and 66 female adult subjects, with no clinical symptoms of disease and with results of the complete blood count and biochemical iron metabolism markers within reference ranges. 

A group of 132 IDA patients fulfilled traditional diagnostic criteria for iron deficiency anemia diagnosis, serum iron < 7.5 *μ*mol/L, transferrin saturation < 20%, ferritin < 50 *μ*g/L, and Hb < 110 g/L, were included before iron treatment. 

This group was divided into a nonacute phase response group (*n* = 72, CRP < 5 mg/L) and acute phase response group (*n* = 60, CRP > 5 mg/L). Acute phase response included inflammation or infectious conditions, in addition to ferropenic status.

CKD patients were managed according to the recommendations of the NKF-K/DOQI guidelines [21]. All patients were treated with a variety of erythropoietin doses, the majority of them were treated with a maintenance dose of intravenous iron weekly, in order to maintain Hb at the recommended level 110–120 g/L.

ACD group included patients with a variety of diseases: chronic infections (tuberculosis); neoplasic disorders (Hodgkin`s disease, breast carcinoma); noninfectious inflammatory diseases (rheumatoid arthritis, systemic lupus erythematosus).

ACD patients received treatment to maintain normal erythropoiesis and presented the traditional diagnostic criteria for “Functional iron-deficiency” diagnosis Transferrin saturation < 20%, Hb < 110 g/L, and serum ferritin values normal or over the reference range.

Table 1 shows the hematological and biochemical data of the different groups. 

### 2.2. Statistical Evaluation of Analytical Results

Statistical software package SPSS (SPSS; Chicago, IL, USA) version 17.0 for Windows was applied for statistical analysis of the results.

Reference ranges were calculated from the results obtained in the group of healthy subjects (95 central percentiles of the distribution). Kolmogorov—Smirnoff test was applied to verify the Gaussian distribution of RSf and LHD% values.

When the parameters under study presented a Gaussian distribution correlation, coefficients were calculated by Pearson method; independent samples *t*-test was performed in order to detect statistical deviations between the groups of patients; *P* values less than  .05 were considered to be statistically significant.

When the parameters under study presented a non-Gaussian distribution correlation, coefficients were calculated by Spearman method; independent samples Mann-Whitney *U*-test was performed; *P* values less than  .05 were considered to be statistically significant.

Receiver operating characteristic (ROC) curve analysis was utilized to illustrate the diagnostic performance of RSf and LHD% in the detection of iron deficiency status, defined by a CHr value 28 pg.

Cutoff values were established based on the optimal combination of sensitivity and specificity.

## 3. Results

**Figure 1:**
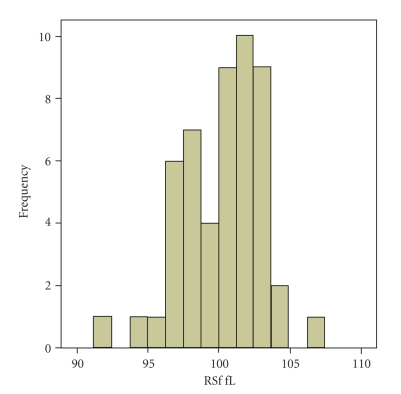
Red blood cell size factor (RSf) values in a population of 120 healthy adult subjects. The values showed Gaussian distribution (Kolmogorov-Smirnoff test, *P* =  .279).

The values of RSf were normally distributed (*P* =  .279) (Figure 1). Reference range was 91.1–106.9 fL.

**Figure 2:**
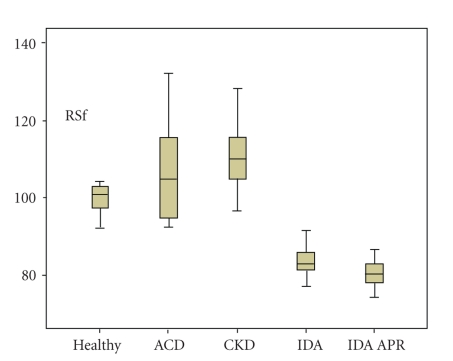
Comparison of red blood cell size factor (RSf) in the groups of anemic patients, anemia of chronic disease (ACD), chronic kidney disease (CKD), iron deficiency anemia (IDA) iron deficiency anemia and acute phase response (IDA APR), and in the healthy group.

Figure 2 and Table 2 exhibit CHr and RSf mean values and standard deviation (SD) in the variety of anemia and healthy subjects included in the study.

The horizontal line in the center of the box shows the median value, the upper and lower limits of the box show the interquartile range, and the whiskers show the minimum and maximum values for each group.

The horizontal line in the center of the box shows the median value, the upper and lower limits of the box show the interquartile range, and the whiskers show the minimum and maximum values for each group.

Correlation between CHr and RSf values, Pearson regression coefficient 0.85, *P *<  .001.

Independent samples *t*-test was performed in order to detect statistical deviations between the groups of patients.

Significant differences in RSf mean values (*P *<  .001) were detected when groups with iron restricted erythropoiesis (IDA, mean 88.1 fL and IDA with APR, mean 84.7 fL) were compared with patients undergoing therapy (ACD, mean 108.9 fL and CKD, 110.6 fL) and the healthy subjects (mean 100.9 fL).

No statistic difference was found between IDA group and IDA patients with acute phase response (*P* =  .481).

IDA and IDA with APR groups presented iron restricted erythropoiesis as is stated by CHr values (24.5 pg and 25.6 pg, resp.) ACD and CKD patients receiving therapy maintained CHr levels higher than the cutoff value of 28 pg.

ROC curve analysis for RSf in the diagnosis of restricted erythropoiesis, defined by CHr < 28 pg, results were AUC 0.983, Cutoff 91.1 fL, sensitivity 98.8%, specificity 89.6%. 

**Figure 3:**
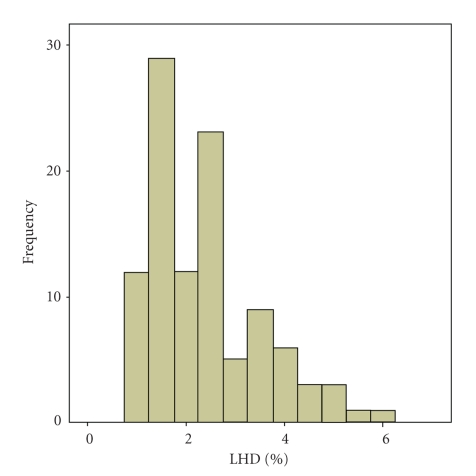
low hemoglobin density (LHD%) values in a population of 120 healthy adult subjects. The values showed a non-Gaussian distribution (Kolmogorov-Smirnoff test, *P *=  .034).

The values of LHD% were not normally distributed (*P* = .034) (Figure 3). Reference range was 0%–4.4%.

**Figure 4:**
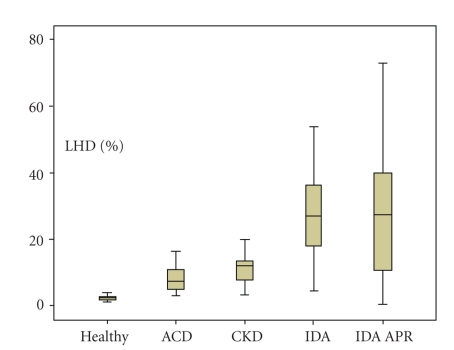
Comparison of low hemoglobin density (LHD%) in the groups of anemic patients, anemia of chronic disease (ACD), chronic kidney disease (CKD), iron deficiency anemia (IDA) iron deficiency anemia and acute phase response (IDA APR), and in the healthy group.

Figure 4 and Table 3 exhibit %Hypo values, mean and standard deviation (SD) and LHD% values, median and 5th–95th interquartiles, in the variety of anemias and healthy subjects included in the study.

The horizontal line in the center of the box shows the median value, the upper and lower limits of the box show the interquartile range, and the whiskers show the minimum and maximum values for each group.

Correlation between %Hypo and LHD% values, *r* = 0.869 (Spearman method) (*P *<  .001). *y* = 1.338*x* + 4.40.

Independent samples *U*-test was performed in order to detect statistical deviations between the groups of patients.

Significant differences in LHD% values (*P *<  .001) were detected when groups with iron deficiency (IDA, mean 29.6% and IDA with APR, mean 27.3%) were compared with patients undergoing therapy (ACD, mean 7.3%; CKD, mean 9.6%) and the healthy subjects (median 2.1%).

No statistic difference was found between IDA group and IDA patients with acute phase response (*P *=  .578).

Receiver operating characteristic (ROC) curve analysis for LHD% in the diagnosis of iron deficiency, defined by %Hypo > 5% AUC 0.954, cutoff 6.0%, sensitivity 96.6%, specificity 83.3%. 

**Figure 5:**
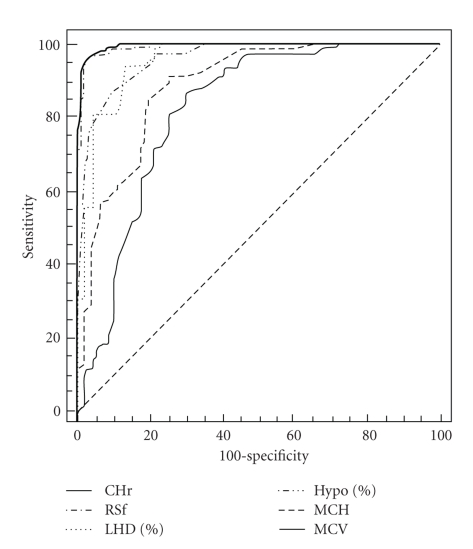
Receiver operating characteristic (ROC) curves for red blood cell size factor (RSf), low hemoglobin density (LHD%), reticulocyte hemoglobin content (CHr), percentage of hypochromic red cells (% Hypo), mean cell hemoglobin (MCH) and mean cell volume (MCV) in the diagnosis of iron deficiency, defined by CHr < 28 pg.

Discriminant efficiency of biochemical parameters and classical erythrocyte indices:

mean cell hemoglobin (MCH), AUC 0.89; mean cell volume, (MCV), AUC 0.822; serum ferritin, AUC 0.722; serum iron, AUC 0.683 (Figure 5).

## 4. Discussion

Uncomplicated iron deficiency is not difficult to diagnose by means of the traditional laboratory tests. Biochemical markers are reliable parameters to diagnose iron deficiency in an uncomplicated clinical setting.

Serum ferritin, as a potent positive acute phase reactant is often increased in ACD and CKD patients [22]. Transferrin saturation is less than 15% to 20% in iron deficiency, but expression of serum transferrin is reported to be downregulated by inflammatory cytokines, so may not reliably reflect iron deficiency when anemia is complicated by inflammation [23].

Efforts have been made to evaluate some readily available and relatively inexpensive laboratory parameters as indirect markers of iron restricted erythropoiesis and iron availability in a clinical context influenced by inflammation and acute phase reaction. 

The best combination of hematological indices for iron deficiency is an increased percentage of hypochromic erythrocytes and a reduced hemoglobin content of reticulocytes [24]. 

Measurements of reticulocyte hemoglobin content (CHr) have been shown to provide useful information in diagnosing functional iron deficiency during r-HuEPO therapy [25, 26] and response to iron therapy [27, 28].

Recent studies confirm the clinical reliability of mature erythrocyte parameters, such as hypochromic red cells (Hypo %), as markers of iron deficiency in hemodialysis patients [29, 30].

Beckman Coulter (Beckman Coulter Inc. Miami, Fl, USA) applies the Volume Conductivity Scatter technology to this field and new parameters are now available on the LH series analysers.

The main purpose of the present study was to determine the diagnostic performance of RSf and LHD% and to assess whether the new parameters correlate with the existing diagnostic tests CHr and %Hypo for restricted erythropoiesis and functional iron deficiency diagnosis.

This study shows a reasonable level of agreement between RSf and CHr. 

Despite the fact that CHr is a measurement of the reticulocyte hemoglobin content and RSf is a measurement of both mature red cells and reticulocyte size, a high concordance between these parameters has been observed and values of both parameters showed the same trend in the different groups.

In patients with iron restricted erythropoiesis, (IDA and IDA APR) RSf values were lower than the reference range 91.1 fL.

ACD and CKD patients were receiving therapy, they suffered a mild anemia but maintained bone marrow activity, as the CHr values state (30.8 and 31.6 pg, resp.). RSf values in these patients were in the reference range of RSf, but the macrocytosis associated to CKD and hemodialysis treatment causes that 15% of the patients in this group had RSf values slightly over the reference range, 106.6 fL.

Diagnostic sensitivity, specificity, and efficiency of RSf were good when compared to a 28 pg value of CHr. In particular, a cutoff value of 91.1 fL for RSf showed the best diagnostic efficiency, with a sensitivity of 98.8% and a specificity of 89.6%. 

This study shows a reasonable agreement between % Hypo and LHD% and their values showed the same trend in the different groups.

%Hypo values in patients undergoing therapy were near the threshold of iron deficient erythropoiesis 5% (ACD 4.1%, CKD 5.1%).

The LHD% values obtained in these groups of patients were statistically lower (ACD 7.3%, CKD 9.6%) than the iron deficient ones (29.6%, 27.3%) but, as all of them had a mild anemia, LHD% values in these patients were above the reference range 4.4%.

The optimal cutoff point for LHD% was 6.0%, which provided sensitivity 96.6%, specificity 83.2%, and area under curve 0.954, for iron deficiency detection, defined by %Hypo > 5%.

In this study, it has been stated that RSf and LHD% could be reliable parameters for the study of iron status and the amount of the supply available for erythropoiesis.

Iron metabolism is a dynamic process which cannot be defined by one test parameter only. The analysis of these new parameters can be performed simultaneously in the course of routine blood counts, with no incremental costs and no additional needs of more blood sampling. In conjunction with standard blood cell counts and iron, parameters could enable the diagnosis to be made rapidly and accurately. 

The new parameters derived from Beckman-Coulter technology seem to be an acceptable alternative to CHr and %Hypo in the routine practice, with the same clinical meaning, but more prospective and longitudinal studies are needed in order to verify the results obtained, to determine their reliability for clinical purposes or whether the additional information provided could be used in managing the iron requirements of patients.

## Figures and Tables

**Table 1:** Hematological and biochemical data of the healthy group (*n* = 120), iron deficiency anemia (IDA, *n* = 72), iron deficiency anemia and acute phase response (IDA APR, *n* = 60), anemia of chronic disease (ACD, *n* = 58), and chronic kidney disease (CKD, *n* = 71) patients.

	RBC 10^12^/L	Hb g/L	MCV fL	MCH pg	MCHC g/L	Iron *μ*mol/L	Transf g/L	Ferritin *μ*g/L	Sat %
Healthy mean	4.9	154	91.1	31.3	343	16.5	2.53	75	31
(SD)	(0.27)	(6.4)	(2.55)	(1.53)	(5.2)	1(0.62)	(0.2)	(2.8)	(1.9)

IDA mean	4.6	95	70	22.5	320	4.8	3.31	14	6
(SD)	(0.61)	(14.2)	(10.3)	(4.23)	(17.3)	(2.15)	(0.53)	(9)	(3.6)

IDA APR mean	4.4	96	75.8	21.5	327	5.1	2.78	37	9
(SD)	(0.43)	(12.1)	(3.7)	(1.3)	(9.2)	(3.5)	(0.28)	(25)	(5.6)

ACD mean	3.5	101	93.2	31.9	343	10.0	2.68	522	15
(SD)	(0.48)	(11)	(6.0)	(2.23)	(10)	(6.8)	(0.66)	(704)	(5)

CKD mean	3.5	112	95.6	31.1	325	9.8	1.87	335	21
(SD)	(0.45)	(8.5)	(6.67)	(2.23)	(8)	(4.47)	(0.43)	(204)	(10)

RBC, red blood cells; Hb, hemoglobin; MCV, mean cell volume; MCH, mean cell hemoglobin; MCHC, mean cell hemoglobin concentration; Transf, transferrin; Sat, % transferrin saturation.

**Table 2:** Reticulocyte hemoglobin content (CHr) and red blood cell size factor (RSf) values, mean and standard deviation (SD), in the group of patients.

	CHr pgmean (SD)	RSf fLmean (SD)
Healthy	33.2 (1.6)	100.9 (5.3)
IDA	24.5 (4.4)	88.1 (7.8)
IDA APR	25.6 (2.5)	84.7 (4.1)
ACD	30.8 (5.1)	105.5 (10.9)
CKD	31.6 (3.5)	110.6 (8.7)

**Table 3:** Percentage of hypochromic red cells (% Hypo) values, mean and standard deviation (SD) and low hemoglobin density (LHD%) values, median (5th–95th interquartiles, IQ) in the group of patients.

	% Hypo Mean (SD)	LHD% Median (IQ)
Healthy	0.13 (0.15)	2.1 (0.9–4.1)
IDA	17.2 (17.4)	29.6 (7.5–76)
IDA APR	16.8 (15.5)	27.3 (8.3–71.2)
ACD	4.1 (4.4)	7.3 (5.1–30)
CKD	5.1 (6.7)	9.6 (5.6–27)
